# The impact of the affordable care act and Medicaid expansion on colorectal cancer screening: Evidence from the 5th year of Medicaid expansion

**DOI:** 10.1002/cam4.7054

**Published:** 2024-04-09

**Authors:** Michael A. Preston, Mahmoud Manouchehri Amoli, Askar S. Chukmaitov, Alex H. Krist, Bassam Dahman

**Affiliations:** ^1^ School of Population Health, Department of Health Behavior and Policy Virginia Commonwealth University Richmond Virginia USA; ^2^ Massey Cancer Center, Health Equity and Disparities Research Virginia Commonwealth University Richmond Virginia USA; ^3^ Department of Pharmacy Practice Purdue University West Lafayette Indiana USA; ^4^ Department of Family Medicine and Population Health Virginia Commonwealth University Richmond Virginia USA

**Keywords:** colorectal cancer screening, health policy, health services research, low‐income, Medicaid expansion, preventive health services

## Abstract

**Background:**

Colorectal cancer screening rates remain suboptimal, particularly among low‐income populations. Our objective was to evaluate the long‐term effects of Medicaid expansion on colorectal cancer screening.

**Design, Setting, and Participants:**

This cross‐sectional study analyzed data from 354,384 individuals aged 50–64 with an income below 400% of the federal poverty level (FPL), who participated in the Behavioral Risk Factors Surveillance System from 2010 to 2018. A difference‐in‐difference analysis was employed to estimate the effect of Medicaid expansion on colorectal cancer screening. Subgroup analyses were conducted for individuals with income up to 138% of the FPL and those with income between 139% and 400% of the FPL. The effect of Medicaid expansion on colorectal cancer screening was examined during the early, mid, and late expansion periods.

**Main Outcomes and Measures:**

The primary outcome was the likelihood of receiving colorectal cancer screening for low‐income adults aged 50–64.

**Results:**

Medicaid expansion was associated with a significant 1.7 percentage point increase in colorectal cancer screening rates among adults aged 50–64 with income below 400% of the FPL (*p* < 0.05). A significant 2.9 percentage point increase in colorectal cancer screening was observed for those with income up to 138% the FPL (*p* < 0.05), while a 1.5 percentage point increase occurred for individuals with income between 139% and 400% of the FPL. The impact of Medicaid expansion on colorectal cancer screening varied based on income levels and displayed a time lag for newly eligible beneficiaries.

**Conclusions:**

Medicaid expansion was found to be associated with increased colorectal cancer screening rates among low‐income individuals aged 50–64. The observed variations in impact based on income levels and the time lag for newly eligible beneficiaries receiving colorectal cancer screening highlight the need for further research and precision public health strategies to maximize the benefits of Medicaid expansion on colorectal cancer screening rates.

## Graphical abstract

Using quasi‐experimental methods, we find that Medicaid expansion was linked to a significant increase in CRC screening rates with incomes below 400% federal poverty level (FPL); specifically, with incomes up to 138% FPL. The effect of Medicaid expansion was not uniform across income levels, and a time lag was observed for newly eligible beneficiaries to receive screening. Policymakers and researchers should consider the time lag and income‐based differences when evaluating the effect of Medicaid expansion on CRC screening rates.

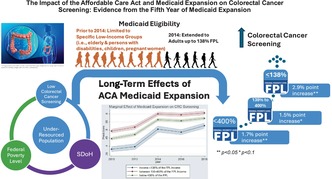

## 1 INTRODUCTION

Colorectal cancer (CRC) is s a significant public health challenge in the United States, representing a major cause of morbidity and mortality.^1^, ^2^ Despite advances in screening techniques and a notable decline in mortality rates, CRC remains the third most common cancer diagnosis and a leading cause of cancer‐related deaths.^3^, ^4^, ^5^ CRC is preventable and treatable.^6^ Cancer screening potentially enables early detection and may increase the probability of improved treatment and survival. CRC screening has substantial net benefits in reducing CRC mortality.^1^, ^7^ The US Preventive Services Task Force (USPSTF) underscores the importance of routine screening for average‐risk adults to improve treatment outcomes and survival rates.^8^, ^9^ However, the current screening rates fall short of national targets set by the National Colorectal Roundtable^10^ and Healthy People 2030,^11^ indicating a gap in preventive healthcare service utilization.

Health insurance coverage plays a pivotal role in screening uptake, with disparities in access and outcomes particularly pronounced among uninsured and low‐socioeconomic‐status individuals.^12^ Lack or inadequate health insurance coverage and unaffordable out‐of‐pocket costs are considered barriers to receiving cancer screening.^13^, ^14^ The Affordable Care Act (ACA), with its Medicaid expansion provision, aimed to address these disparities by increasing insurance coverage among low‐income adults with an income up to 138% of the federal poverty level (FPL) in states that chose to participate. For those at an income level between 100% and 400% of the FPL, the ACA provides subsidies to purchase health insurance. While evidence suggests that the ACA has improved access to care and CRC screening rates, findings remain mixed, with some studies indicating no change and others reporting increases in screening uptake, particularly in early expansion states.

Several studies confirmed that Medicaid expansion improved insurance coverage^15^, ^16^ and access to primary care physicians in both rural and urban areas in expansion states compared with non‐expansion states.^17^, ^18^, ^19^, ^20^, ^21^ By removing cost‐sharing, the ACA made preventative services such as CRC screening more accessible to Americans.^22^ Moreover, increasing coverage through Medicaid expansion is critical for increasing colorectal cancer screening rates.^6^ Several studies have suggested that Medicaid expansion is related to increasing CRC screening and early diagnosis.^7^, ^13^, ^23^, ^24^ However, other studies found insignificant effects of Medicaid expansion on screening tests.^25^, ^26^ For instance, studies have found that Medicaid expansion caused no change in CRC screening in community health centers^27^ or CRC screening rates increased in early expansion states and not in the states that expanded Medicaid after 2014.^07^

Considering these mixed results, our study aims to build upon the existing knowledge by leveraging the Behavioral Risk Factor Surveillance System (BRFSS) to analyze the longitudinal effects of Medicaid expansion on CRC screening behaviors. Specifically, we employ an income‐stratified difference‐in‐difference (DD) model to elucidate the long‐term impact of this policy change and to investigate, whether the benefits of Medicaid expansion are uniformly experienced across income levels and time in the recommended age group (50–64 years old). This nuanced analysis is critical to inform targeted interventions and policy efforts that enhance CRC screening rates, particularly among populations that traditionally face barriers to accessing preventive care.

## 2 METHODS

### 2.1 Data source

Data from 2010 to 2018 from the BRFSS was used to measure the effect of Medicaid expansion on CRC screening rates. BRFSS is a publicly available annual telephone survey conducted by the Center for Disease Control and Prevention (CDC) in collaboration with state health departments since 1984. As a federally funded and nationally representative survey, BRFSS collects individual‐level data from more than 400,000 survey responders with state identifiers, which allowed us to determine whether respondents resided in expansion or non‐expansion states.

BRFSS has information about annual household income within eight categories. To calculate individual income as a percent of the FPL, we obtained states' poverty guidelines from the Department of Health and Human Service.^28^ Lastly, using the midpoint method,^29^ we derived continuous individual income. The data about Medicaid expansion status was obtained from Kaiser Family Foundation.^30^

### 2.2 Study population

Our final analytical sample consisted of 354,384 adults aged 50–64 with income below 400% of the FPL, who had answered CRC screening questions in BRFSS from 2010 to 2018. We excluded Wisconsin because it did not expand Medicaid but gave the benefits to adults below 100% of the FPL, so it acts more like an expansion state. Also, we excluded observations with missing values on income, age, sex, race, education, and employment status.

### 2.3 Outcome variable

The outcome of interest was self‐reported CRC screening. Recommended by USPSTF, CRC screening options for average‐risk adults aged 50 include fecal immunochemical test (FIT) or fecal occult blood test (FOBT) every year, stool‐DNA every 3 years, colonoscopy every 10 years, and flexible sigmoidoscopy every 5 years with FIT every year.^31^ The BRFSS has a calculated variable (crcrec) to determine whether respondents have received recommended CRC screening based on their response to biannual questions about receiving FOBT, sigmoidoscopy, or colonoscopy in 2014, 2016, and 2018. A similar variable was created for the CRC screening in 2010 and 2012. We coded the dependent variable as a dichotomous dummy variable equal to the one for people who reported having received recommended CRC screening and zero otherwise (see Table S1).

### 2.4 Empirical model

We used a DD model to examine the effect of Medicaid expansion on CRC screening, comparing expansion, and non‐expansion states before and after Medicaid expansion. The expansion states consist of 31 states and the District of Columbia (D.C.) that expanded the Medicaid program between 2014 and 2018 (see Table S2). These were considered to be the treatment group. The non‐expansion states were considered the control group. Because Medicaid expansion was implemented in different years across states, we used a DD approach used by Soni^32^ to estimate the effect of Medicaid expansion. Using the variation on when states expanded the Medicaid program, we estimate the following DD model:

$${\text{Screen}}_{\mathrm{ist}}=\alpha +\beta {\text{Expansion}}_{\mathrm{st}}+\gamma X_{\mathrm{ist}}+\omega {\text{State}}_{s}+\theta {\text{Year}}_{t}+\varepsilon_{\mathrm{ist}}$$

where ${\text{Screen}}_{\mathrm{ist}}$ represents CRC screening as the main outcome of interest and is a binary variable for receiving CRC screening, for individual *i*, in the state *s*, and at the time *t*. ${\text{Expansion}}_{\mathrm{st}}$ is a binary indicator equal to 1 if the individual lives in an expansion state and 0 if the respondent resides in a non‐expansion state or has been interviewed before the implementation of Medicaid expansion. $X_{\mathrm{ist}}$ is a vector of individual‐level characteristics including, age, sex, race, marital status, education, income level, health status, and whether responders landline or cellphone users. The ω and θ are capturing state‐ and time‐fixed effects. State and year‐fixed effects allow us to control for time‐invariant state characteristics and to capture temporal changes. β captures the Medicaid expansion effect. This effect assumes that expansion and non‐expansion states are expected to have similar outcome trends in the absence of the intervention.

We also used a second model to estimate the Medicaid expansion effect on CRC screening since the time of expansion:

$$\begin{array}{cc} {\text{Screen}}_{\mathrm{ist}}= & \alpha +\sum_{j=1}^{5}\beta_{j}D_{s}1\left(\;t-\text{ExpansionYear}+1=j\right)\\ & +\gamma X_{\mathrm{ist}}+\omega {\text{State}}_{s}+\theta {\text{Year}}_{t}+\varepsilon_{\mathrm{ist}} \end{array}$$

where the index of *j* indicates the time since implementation of Medicaid expansion for each expansion state. In BRFSS, the CRC screening questions have been asked biannually. Thus, *j* has been defined as 2‐year periods, such that the first 2 years after expansion are considered to be early expansion years, the second 2 years after expansion are considered to be middle (mid) expansion years, and the 5th year after expansion is considered to be the late expansion years. The DD estimate of the Medicaid expansion effects on CRC screening has been captured by $\beta_{j}$. This approach enables us to separate the expansion effects in secular periods following Medicaid expansion.

We delineate the timing of Medicaid expansion effects by categorizing the years post‐expansion into distinct periods. For instance, for the states expanded in 2014, early expansion years would be years 1 and 2 post‐expansion, referring to 2014–2015, middle expansion years consists of years 3 and 4, referring to 2016–2017, and late expansion year is year 5, referring to 2018. This stratification allows us to capture the immediate and delayed effects of policy implementation on CRC screening behaviors among low‐income individuals. Our approach echoes the methodology applied in other studies assessing policy impacts over time, thereby ensuring that our analysis remains aligned with the established research protocols for longitudinal policy evaluation.

Moreover, income stratified analysis allowed us to assess whether low‐income individuals benefited from Medicaid expansion. We categorized participants into groups based on their income relative to the FPL, with particular focus on those up to 138% of the FPL—the primary beneficiaries of Medicaid expansion—and those between 139% and 400% of the FPL who are eligible for subsidized health insurance coverage.

For the sensitivity analysis, because BRFSS lacks the specification of type of insurance coverage such as Medicaid, Medicare, or commercial, we utilized age as a natural proxy for Medicare eligibility. We used the lower‐income adults aged 65–75 who are subject to Medicare (see eSensitivity Analysis in the Supplement—Data S1). The parallel trend assumption is crucial in DD designs, as it ensures that the treatment and control groups (expansion and non‐expansion states) would have followed similar trends in CRC screening rate in the absence of intervention (Medicaid expansion). However, the parallel trend assumption cannot be directly tested, and in our study, we faced the challenge of time‐varying treatment (expansion). To address this issue, we employed an approach used in previous research by performing an event study plot as a substitute for the parallel trend assumption.^33^ This method, which has been utilized in similar investigations,^34^, ^35^ allowed us to visually assess the plausibility of the parallel trends assumption,^36^ providing additional robustness to our findings and bolstering the causal interpretation of the Medicaid expansion's impact on CRC screening rates (see eEvent Study Plots in the Supplement—Data S1). By using the event study plot, we were able to account for the time‐varying nature of the Medicaid expansion and further strengthen the validity of our analysis.

The standard error for our analysis clustered at the state level, and we used BRFSS individual sampling weights for all analyses. The analysis was performed using the Stata package, and Ordinary Least Squares were used for our estimations.

## 3 RESULTS

The sociodemographic characteristics of the study population who are screened for CRC are presented in Table 1. Of the 354,384 survey respondents, 202,027 (57%) had been screened for CRC and 152,357 (43%) had not been screened for CRC.

**TABLE 1:** Demographic characteristics of adults aged 50–64 with income below 400% of FPL, BRFSS 2010–2018.

	Total	Non‐screened for CRC				Screened for CRC			
		Total non‐screened	Medicaid expansion states	Non‐Medicaid expansion states	*p* value	Total screened	Medicaid expansion states	Non‐Medicaid expansion states	*p* value
*N*	354,384	152,357	46,838 (30.7%)	105,519 (69.3%)	–	202,027	80,444 (39.8%)	121,583 (60.2%)	–
	(%)	(%)	(%)	(%)		(%)	(%)	(%)	
Sex									
Male	47.9	49.8	49.8	49.8	0.99	46.5	47.1	46.0	0.024
Female	52.1	50.2	50.2	50.2		53.5	52.9	54.0	
Race/ethnicity									
Whites NH	67.6	64.6	65.3	64.2	<0.00	69.8	70.3	69.3	<0.00
Blacks NH	13.3	12.5	9.5	14.2		14.0	10.9	16.7	
Hispanic	12.4	15.7	16.1	15.5		9.8	11.0	8.9	
Others NH	6.7	7.2	9.1	6.1		6.4	7.8	5.1	
Marital status									
Married	62.9	58.7	60.3	57.7	<0.00	66.1	66.9	65.5	<0.00
Non‐married	37.1	41.3	39.7	42.3		33.9	33.1	34.5	
Employment									
Employed	58.5	61.4	64.0	59.8	<0.00	56.2	59.1	53.7	<0.00
Non‐employed	41.5	38.6	36.0	40.2		43.8	40.9	46.3	
Education									
Less than high school	14.6	19.0	17.5	19.9	<0.00	11.2	10.8	11.5	<0.00
High school degree	30.9	32.6	32.0	33.0		29.7	28.7	30.6	
Some college	31.0	29.0	29.2	28.8		32.5	32.3	32.7	
College degree	23.5	19.4	21.3	18.3		26.6	28.2	25.2	
Health insurance coverage									
Yes	86.5	77.5	86.4	72.0	<0.00	93.4	95.6	91.5	<0.00
No	13.5	22.5	13.6	28.0		6.6	4.4	8.5	
Access to personal doctor									
Have personal doctor	85.7	75.4	78.8	73.8	<0.00	93.6	94.1	93.2	0.00
Do not have personal doctor	14.3	24.6	22.0	26.2		6.4	5.9	6.8	
Health status									
Excellent	13.9	14.4	15.2	13.9	<0.00	13.5	14.1	12.9	<0.00
Very good	27.6	26.4	27.8	25.5		28.5	30.1	27.1	
Good	31.9	32.5	32.4	32.6		31.5	30.8	31.8	
Fair	18.1	18.4	17.6	18.8		18	17.5	18.5	
Poor	8.5	8.3	7.0	9.2		8.7	7.5	9.7	

Abbreviation: NH, non‐Hispanic.

DD estimates for CRC screening after the implementation of the ACA Medicaid expansion showed that Medicaid expansion was associated with a significant 1.7 percentage point increase in CRC screening for the people with income below 400% of the FPL in expansion states compared with non‐expansion states (Table 2). A significantly higher increase in CRC screening (2.9 percentage points) was observed in lower‐income individuals with an income up to 138% of the FPL compared to individuals with an income between 139% and 400% of the FPL, who showed an increase of 1.5 percentage points in CRC screening rates (Table 2).

**TABLE 2:** Difference‐in‐difference estimates of the impact of the ACA Medicaid expansion on colorectal cancer screening for adults aged 50–64 with income below 400% of FPL, 2010–2018.

Income level	Expansion effect on CRC screening DD estimates (std. err.)	95% CI
<400% FPL	0.017 (0.007)**	(0.003, 0.031)
≤138% FPL	0.029 (0.015)**	(0.000, 0.058)
Between 139% and 400% FPL	0.015 (0.008)*	(−0.001, 0.032)

** *p* < 0.05.

* *p* < 0.1.

Overall, for the entire analytical sample consisting of adults aged 50–64 with income below 400%, we found that CRC screening significantly increased by two percentage points in the mid‐expansion years (3rd and 4th years after expansion) (Table 3). We found a similar significant 2% percentage points increase during the late expansion years (5th year after expansion) for this income group. However, our results did not show any significant change in the CRC screening during the early expansion years (1st and 2nd year after implementation of Medicaid expansion) for this group (Table 3).

**TABLE 3:** Difference‐in‐difference estimates of the impact of the ACA Medicaid expansion on colorectal cancer screening for adults aged 50–64 with income below 400% of FPL by the number of years after expansion, 2010–2018.

	DD estimates	*p* value	95% CI
The estimates for adults aged 50–64 with income below 400% of the FPL			
Early‐expansion	0.007 (0.008)	0.392	(−0.009, 0.023)
Mid‐expansion	0.020 (0.009)**	0.033	(0.002, 0.038)
Late‐expansion	0.020 (0.011)*	0.087	(−0.003, 0.042)
The estimates for adults aged 50–64 with income up to 138% of the FPL			
Early‐expansion	0.016 (0.017)	0.361	(−0.018, 0.050)
Mid‐expansion	0.049 (0.017)***	0.005	(0.014, 0.083)
Late‐expansion	0.022 (0.021)	0.279	(−0.018, 0.063)
The estimates for adults aged 50–64 with income between 139% and 400% of the FPL			
Early‐expansion	0.011 (0.009)	0.239	(−0.007, 0.028)
Mid‐expansion	0.014 (0.010)	0.177	(−0.006, 0.034)
Late‐expansion	0.026 (0.013)**	0.041	(0.001, 0.052)

*Note*: Early‐expansion years (1st and 2nd year after expansion). Mid‐expansion years (3rd and 4th years after expansion). Late‐expansion years (5th year after expansion).

*** *p* < 0.001.

** *p* < 0.05.

* *p* < 0.1.

Interestingly, we found that adults with an income up to 138% of the FPL experienced a significant increase of 4.9 percentage points in the mid‐expansion years. For this group, we could not find any significant change in CRC screening during the early or late expansion years. Lastly, for the adults with income between 139% and 400% of the FPL, we only found a significant increase in CRC screening during the late‐expansion period, without finding any statistically significant changes in CRC screening rates during the early or mid‐ expansion years following Medicaid expansion (Table 3).

**FIGURE 1:**
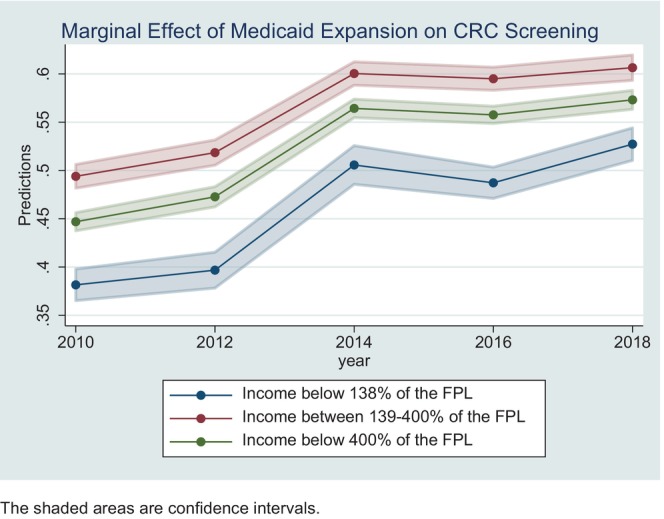
Marginal effect of Medicaid expansion on CRC screening for adults aged 50–64 with income below 400% of FPL, 2010–2018. The shaded areas are confidence intervals.

Figure 1 shows the marginal effects of Medicaid expansion on CRC screening by income level. As shown, the CRC screening rates increased in the post‐ACA period for all income groups. However, individuals with an income up to 138% of the FPL had a lower screening rate than those with an income 139%–400% of the FPL. Even after the implementation of the ACA Medicaid expansion, there is still a gap in CRC screening rates between the two income groups. However, we see some evidence that this gap is shrinking in the late expansion years.

## 4 DISCUSSION

In this cross‐sectional study, a quasi‐experimental DD design was used to evaluate the long‐term effects of Medicaid expansion on CRC screening among low‐income individuals aged 50–64, conducting income‐stratified analyses, and capturing the time lag in the effect of the expansion. Looking at an extended period, we found that Medicaid expansion is associated with increased CRC screening rates. Our study's income stratification elucidates the nuanced effects of Medicaid expansion across different economic segments of the population. This approach highlights the variability in policy impact and underscores the importance of tailoring health interventions to meet the needs of diverse income groups. Moreover, our study sheds light on the time lag for newly eligible beneficiaries to receive CRC screening following the implementation of Medicaid expansion. This aspect has been underexplored in the existing literature, which primarily focuses on the short‐term effects of Medicaid expansion. By examining the early, mid, and late expansion periods, we were able to identify the time lag in the effect of Medicaid expansion on CRC screening rates for different income groups. These findings contribute to a more comprehensive understanding of the effectiveness of Medicaid expansion in increasing access to preventive healthcare services, emphasizing the importance of considering both income‐based differences and the time lag when evaluating the effect of Medicaid expansion on CRC screening rates. By addressing these research gaps, our study provides valuable insights for policymakers and researchers seeking to maximize the benefits of Medicaid expansion and improve CRC screening rates among low‐income populations.

The association between Medicaid expansion and increasing various types of cancer screening is well established.^37^ In accordance with previous research and looking at longer periods after Medicaid expansion, we find that expansion is associated with increased CRC screening rates.^12^, ^38^ Interestingly, in contrast to other studies,^7^, ^23^ we did not find any significant effects during the first two years after expansion. These insignificant effects on CRC screening during the early expansion years have been emphasized previously.^25^, ^27^ Thus, our results help explain the mixed results in some previously published literature. We showed that while CRC screening did not significantly increase during the early years after expansion, Medicaid expansion is associated with increased likelihood of receiving CRC screening for lower‐income individuals.

Notably, our results highlight the lag in the effect of Medicaid expansion on CRC screening for adults in all income groups. This delay could be attributed to many factors, particularly to the characteristics of individuals with different income levels. We observed heterogeneity in the expansion effects based on income level. For instance, for individuals with an income up to 138% of the FPL, a significant increase in CRC screening was observed in the mid‐expansion years. On the other hand, CRC screening significantly increased during the late‐expansion period for adults with income between 139% and 400% of the FPL. Therefore, for the full analytical sample, we can argue that the increase in the mid‐expansion years was attributed to individuals with income up to 138% FPL, while the increase in the late‐expansion years was attributed to individuals with income of 139%–400% FPL.

Our analysis reveals that the impact of Medicaid expansion on CRC screening is not uniform across all income groups or consistent over time. Particularly, we observed a significant increase in CRC screening among adults with incomes up to 138% of the FPL during the mid‐expansion years, suggesting a delayed response to the policy change in this demographic. Conversely, for adults with incomes between 139% and 400% of the FPL, the increase in screening rates was more pronounced in the late expansion period. These findings indicate that while Medicaid expansion broadly contributes to increased CRC screening, the effects are staggered and vary by income level. It highlights the need for targeted strategies that address the specific barriers and facilitators influencing screening behavior in different socioeconomic segments. Additionally, the diminishing effect on the lowest income group in the late expansion period raises important questions about the sustainability and long‐term effectiveness of the policy in increasing screening rates among the most economically vulnerable populations.

Lastly, our findings showed that improving access through expanding health insurance coverage is associated with increased CRC screening, which eventually promotes earlier diagnosis and better care outcomes among patients with CRC.^38^ However, factors such as access to primary care providers can mask the effect of Medicaid expansion,^13^ where higher screening should be expected in states with a higher number of primary care providers. Moreover, it is shown that physician visits moderate preventative visits such as CRC screenings.^6^ It is worth mentioning that insurance coverage is only one of the important factors that help lower‐income individuals receive CRC screening, mainly by reducing out‐of‐pocket costs.^39^ Effective policies are needed to target various barriers that block the paths of receiving timely recommended cancer screenings.

## 5 LIMITATIONS

This study is not without limitations. BRFSS is a cross‐sectional nationally representative survey. Thus, we were not able to follow individuals longitudinally. Moreover, the responses on BRFSS, including CRC screening rates, are self‐reported and therefore subject to response and recall bias. This self‐reporting nature of data collection may lead to an overestimation of screening rates, which is a crucial consideration in interpreting our findings.^40^ Because the BRFSS only asks individuals aged 50–75 CRC screening questions, the screening measures reported in this article do not account for the most recent guideline updates of the USPSTF, which recommend initiating CRC screening at age 45 for average‐risk individuals.^9^ Lastly, the income threshold for each individual in BRFSS is not a clear line. As mentioned in the method section, we categorized individuals with a household income of up to 400% of the FPL using the midpoint method,^29^ which may be debatable in some cases.

## 6 CONCLUSION

In this study, we evaluate whether Medicaid expansion is associated with changes in receiving recommended CRC screening among low‐income adults aged 50–64 years old. We found that Medicaid expansion increased the likelihood of low‐income individuals to receive CRC screening. We also found heterogeneous effects of Medicaid expansion on CRC screening among lower‐income individuals at different income levels. This study highlights the income‐level variation in the impact of Medicaid expansion on CRC screening uptake. Moreover, our finding highlights the importance of monitoring the long‐term effect of Medicaid expansion on CRC screening among the low‐income populations, which help the policymakers to design more targeted and effective interventions.

## AUTHOR CONTRIBUTIONS

**Michael A. Preston:** Conceptualization (lead); data curation (lead); formal analysis (equal); funding acquisition (lead); investigation (equal); methodology (lead); project administration (lead); resources (equal); software (equal); supervision (lead); validation (equal); visualization (equal); writing – original draft (lead); writing – review and editing (lead). **Mahmoud Manouchehri Amoli:** Conceptualization (equal); data curation (equal); formal analysis (equal); investigation (equal); methodology (equal); software (equal); validation (equal); visualization (equal); writing – original draft (equal); writing – review and editing (equal). **Askar S. Chukmaitov:** Investigation (supporting); methodology (supporting); validation (supporting); writing – review and editing (supporting). **Alex H. Krist:** Conceptualization (supporting); investigation (equal); methodology (supporting); resources (supporting); validation (equal); writing – review and editing (supporting). **Bassam Dahman:** Conceptualization (supporting); formal analysis (equal); methodology (equal); software (equal); supervision (equal); validation (equal); visualization (equal); writing – review and editing (supporting).

## FUNDING INFORMATION

This work by Dr. M.A. Preston and the research team was supported by the NCI‐designated VCU Massey Cancer Center Office of Health Equity & Disparities Research with funding from NIH‐NCI Cancer Center Support Grant P30 CA016059; Wright Center's Clinical and Translational Science Award (CTSA), CTSA grant number: KL2TR002648; Institutional Research Grant IRG‐18‐159‐43 from the American Cancer Society; and Geographical Management of Cancer Health Disparities (GMaP) Program in Region 1 North Stimulus Award P30 CA177558. This project would not happen without the support of the Reaching the Underserved, Rural, and Low‐Income (RURaL) Lab for D&I Research in Cancer Disparities and Center for Health Equity and Innovation. Special thanks to Heidi Sankala, PhD, Scientific Writing Manager at Massey Cancer Center, for her technical review; Khalid Matin, MD and Vanessa B. Sheppard, PhD for their critical feedback.

## CONFLICT OF INTEREST STATEMENT

The authors declare no potential conflict of interest.

## ETHICS STATEMENT

No Institutional Review Board approval was necessary prior to commencing this study. This research is considered exempt from ethical approval due to data is publicly available and does not constitute human subjects research under 45. CFR 46. This study does not involve human participants or patient material (tissue/blood samples) and therefore does not require written informed consent.

## Supporting information


Data S1:

## Data Availability

Data sharing is not applicable to this article as no new data were created and all data is publicly available.
