# Supplementary material for: The impact of the affordable care act and Medicaid expansion on colorectal cancer screening: Evidence from the 5th year of Medicaid expansion
Source: Cancer Med. 2024 Apr 9;13(7):e7054. doi: 10.1002/cam4.7054 (PMC11002632; doi:10.1002/cam4.7054)

**eTable 1. Variables Used in this Study**

| **Variable Name** | **Sources of Data** | **Question in BRFSS** | **Variable Type** | **Variable Label** |
| --- | --- | --- | --- | --- |
| **Outcome Variables** | | |  |  |
| **Access Outcomes** | | | | |
| CRC Screening | BRFSS- Core Module  (Biannual) | Respondents aged 50-75 who have fully met the USPSTF recommendation | Binary | Have been Screened for CRC=1, otherwise=0 |
| **Independent Variables** | | | | |
| Expansion | KFF Medicaid Expansion Status | Whether a specific state is expanded the Medicaid program in a specific year | Time varying Binary | Dummy if expanded=1 o.w.=0 |
| **Control Variables** | | |  |  |
| Education | BRFSS- Core Module | Level of education completed | Categorical | Below high school,  High school degree(ref.), Some college, College degree |
| Marital | BRFSS- Core Module | Are you: (marital status) | Categorical | Married(reference) and non-married |
| Age | BRFSS- Core Module | Calculated age category | Categorical | 50-54(reference), (55-59), and (60-64) |
| Race | BRFSS- Core Module | Race/ethnicity categories | Categorical | None-Hispanic (NH) White (ref.), NH Blacks, Hispanics, Others |
| Sex | BRFSS- Core Module | What was your sex at birth? Was it male or female? | Categorical | Male and female(reference) |
| Employment Status | BRFSS- Core Module | Are you currently…? | Binary | If employed=1 o.w.=0 |
| Health Status | BRFSS- Core Module | Would you say that in general your health is: | Categorical | Excellent, very good, good, fair(ref.), poor, unknown |
| **Fixed effects** | | |  |  |
| Year fixed effects | BRFSS- Core Module | Interview Year | Categorical |  |
| State fixed effects | BRFSS- Core Module | State FIPS Code | Categorical |  |

**eTable 2. The Cohorts of Expansion and Non-Expansion States from 2010-2018 by the Year of Expansion Used in Difference-in-Difference Model**

|  | **The cohort of states expanded in 2014** | **The cohort of states expanded in 2015** | **The cohort of states expanded in 2016** | **The cohort of non-expansion states** |
| --- | --- | --- | --- | --- |
|  | Arizona | Alaska | Louisiana | Alabama |
|  | Arkansas | Indiana | Montana | Florida |
|  | California | Pennsylvania |  | Georgia |
|  | Colorado |  |  | Kansas |
|  | Connecticut |  |  | Mississippi |
|  | Delaware |  |  | Missouri |
|  | District of Columbia |  |  | Nebraska |
|  | Hawaii |  |  | North Carolina |
|  | Illinois |  |  | Oklahoma |
|  | Iowa |  |  | South Carolina |
|  | Kentucky |  |  | South Dakota |
|  | Maryland |  |  | Tennessee |
|  | Massachusetts |  |  | Texas |
|  | Michigan |  |  | Wyoming |
|  | Minnesota |  |  | Maine |
|  | Nevada |  |  | Virginia |
|  | New Hampshire |  |  | Idaho |
|  | New Jersey |  |  | Utah |
|  | New Mexico |  |  |  |
|  | New York |  |  |  |
|  | North Dakota |  |  |  |
|  | Ohio |  |  |  |
|  | Oregon |  |  |  |
|  | Rhode Island |  |  |  |
|  | Vermont |  |  |  |
|  | Washington |  |  |  |
|  | West Virginia |  |  |  |

**eSensitivity Analysis. The sensitivity test is looking at the age group 65-75 who are subject to Medicare and screened for CRC**

Results of sensitivity analysis: as is shown in the tables below none of the results are significant, show that the Medicaid expansion is not associated with an increase in CRC screening among adults aged 65-75 who are subjects to Medicare, which confirm our results presented in the manuscript.

**eTable3.** Difference-in-Difference Estimates of The Impact of the ACA Medicaid Expansion on Colorectal Cancer Screening for Adults Aged 65-75 with Income Below 400% of the FPL, 2010-2018

| Income Level | Expansion effect on CRC screening  DD estimates (std. err.) | p-value | 95% CI |
| --- | --- | --- | --- |
| *< 400% FPL* | -0.006 (0.008) | 0.501 | (-0.022, 0.011) |
| *< 138% FPL* | -0.010 (0.020) | 0.615 | (-0.050, 0.030) |
| *between 139-400% FPL* | -0.002 (0.009) | 0.774 | (-0.019, 0.014) |

** p<0.05 * p<0.1

**eTable4.** Difference-in-Difference Estimates of the Impact of the ACA Medicaid Expansion on Colorectal Cancer Screening for Adults Aged 65-75 with Income below 400% of the FPL by the Number of Years after Expansion, 2010-2018

|  | DD estimates | p-value | 95% CI |
| --- | --- | --- | --- |
| *The estimates for adults aged* 65-75 *with income below 400% of the FPL* | | | |
| Early-expansion | -0.002 (0.009) | 0.801 | (-0.020, 0.015) |
| Mid- expansion | -0.002 (0.010) | 0.851 | (-0.022, 0.018) |
| Late-expansion | -0.017 (0.012) | 0.167 | (-0.041, 0.007) |
| *The estimates for adults aged* 65-75 *with income below 138% of the FPL* | | | |
| Early-expansion | -0.014 (0.023) | 0.537 | (-0.060, 0.031) |
| Mid- expansion | -0.011 0.024) | 0.643 | (-0.059, 0.037) |
| Late-expansion | -0.003 (0.029) | 0.919 | (-0.059, 0.053) |
| *The estimates for adults aged* 65-75 *with income* *between 139-400% of the FPL* | | | |
| Early-expansion | 0.002 (0.009) | 0.833 | (-0.016, 0.020) |
| Mid- expansion | 0.002 (0.011) | 0.858 | (-0.019, 0.023) |
| Late-expansion | -0.019 (0.012) | 0.136 | (-0.043, 0.006) |

**eEvent Study Plots.**

**eFigure1.** Event Study Plots by income subgroup

**eFigure2. Event Study Plots by income subgroup and by cohort of expansion**


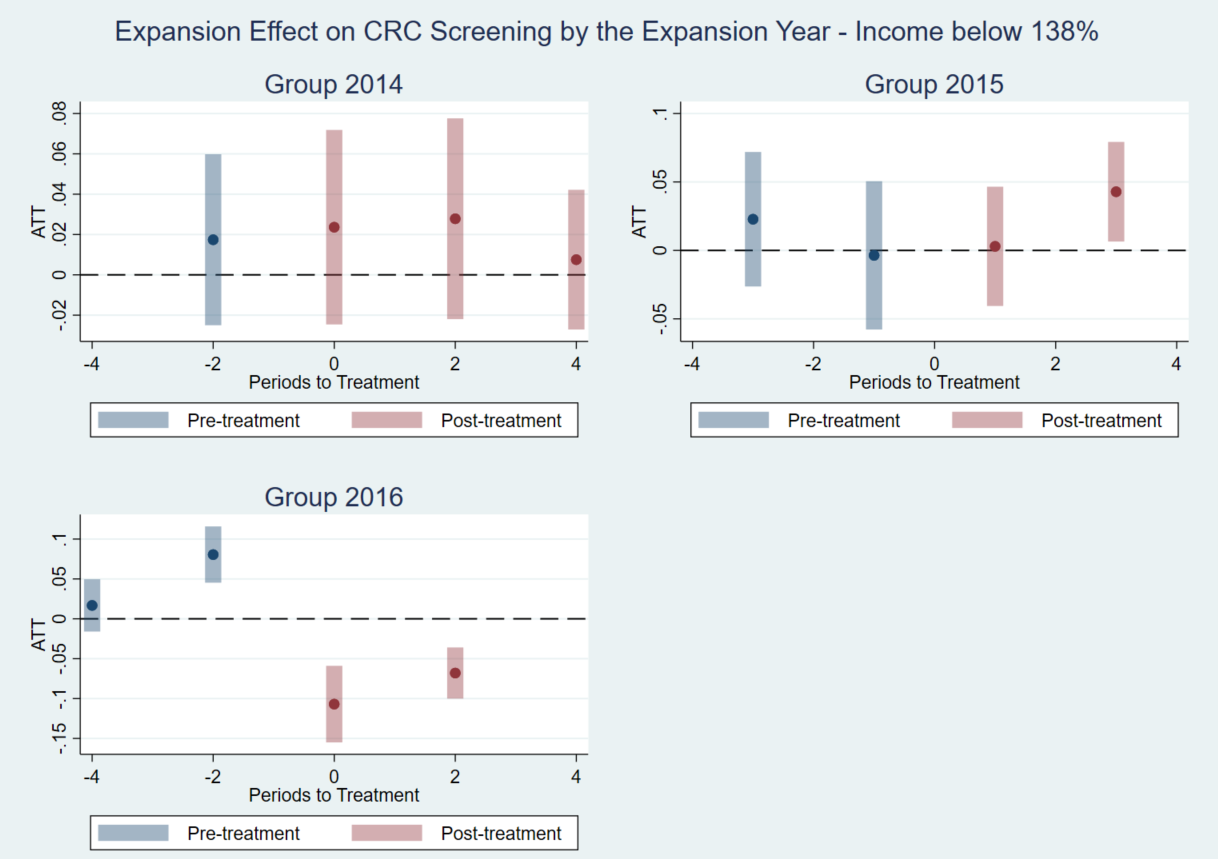


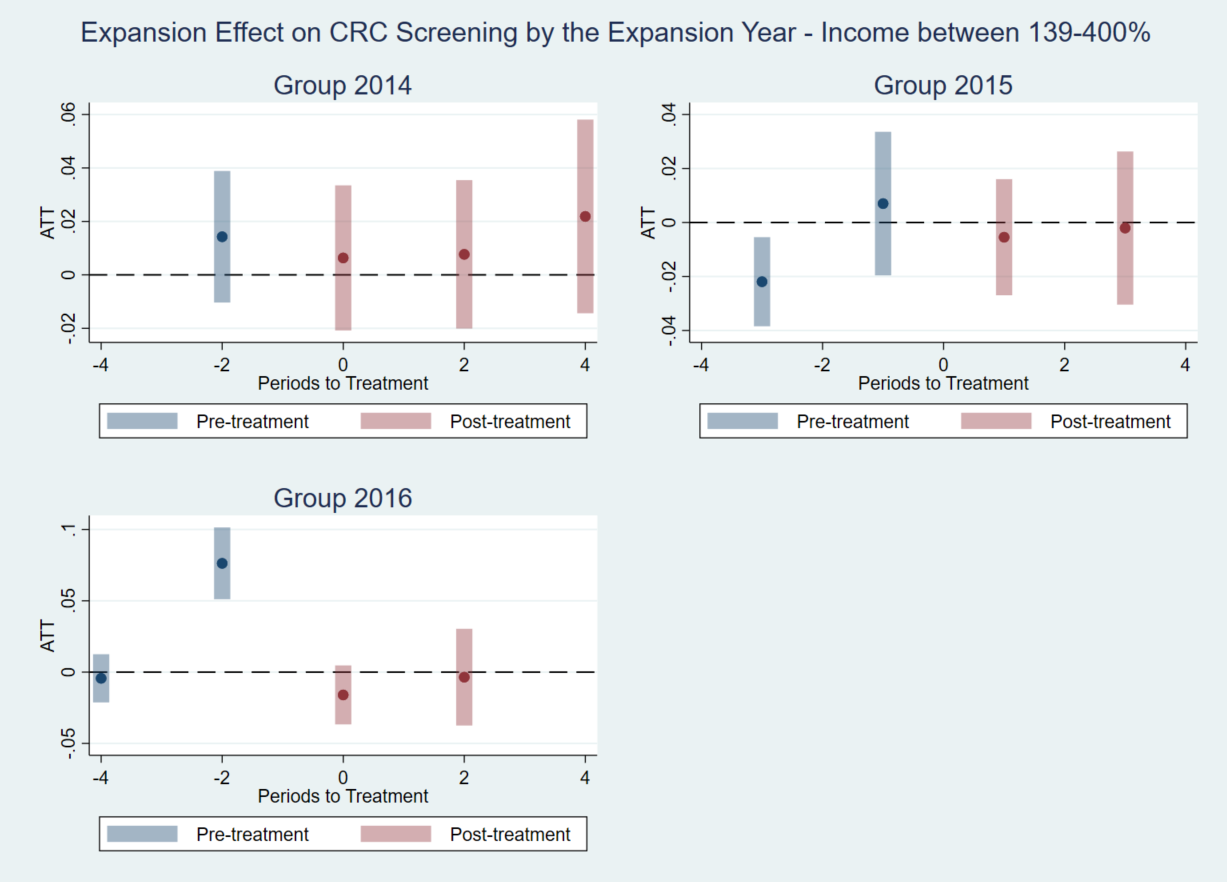


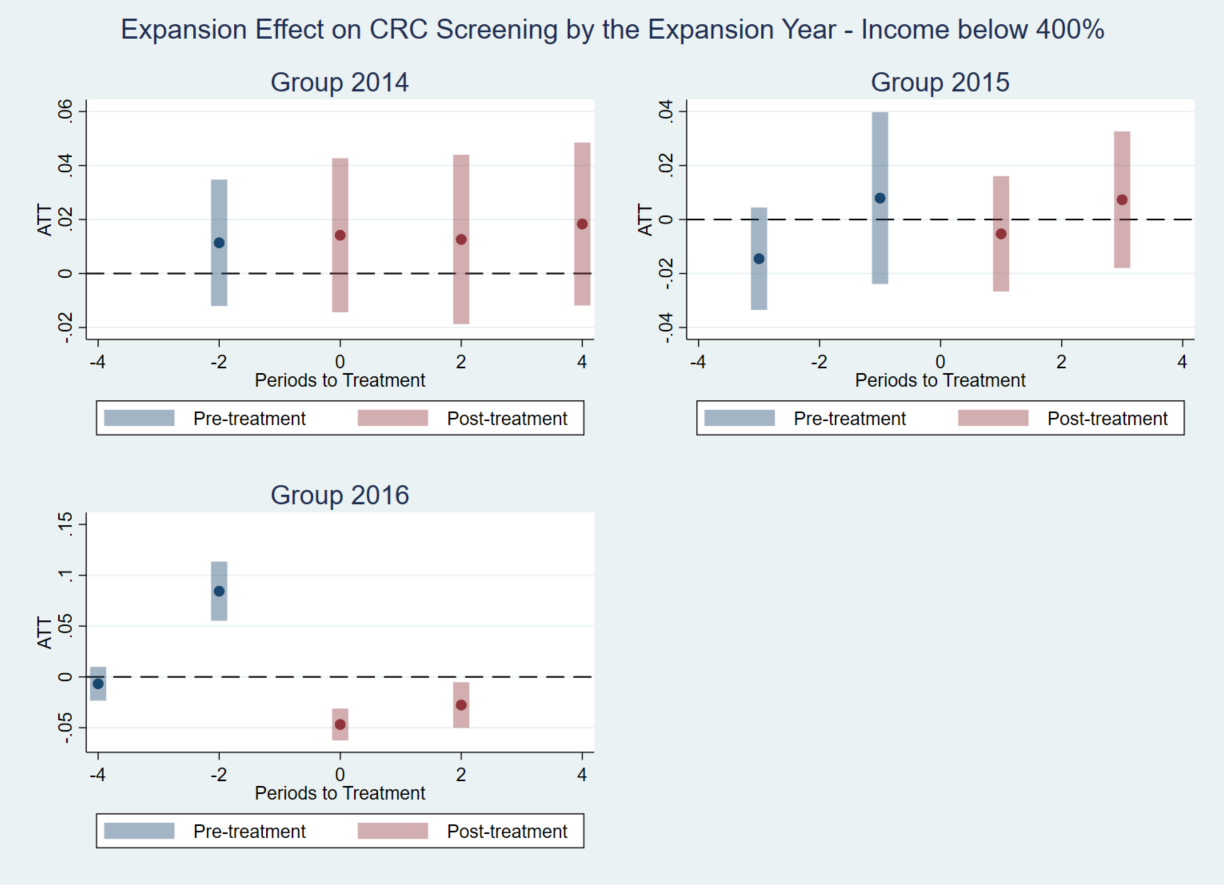

Supplement: Supplementary file 1 — Data S1: [file CAM4-13-e7054-s001.docx]
